# Phylogenetic inconsistency of pairwise SNP clustering for inferring tuberculosis transmission in a high-burden, endemic setting: a case study from Thailand

**DOI:** 10.1128/msystems.00641-26

**Published:** 2026-09-14

**Authors:** Pakorn Aiewsakun, Walter Samuel Hunter, Prasit Palittapongarnpim

**Affiliations:** 1 Department of Microbiology, Faculty of Science, Mahidol University 26685, Bangkok, Thailand; 2 Pornchai Matangkasombut Center for Microbial Genomics, Department of Microbiology, Faculty of Science, Mahidol University 26685, Bangkok, Thailand; University of South Florida Morsani College of Medicine, Tampa, Florida, USA; Northern Arizona University, Flagstaff, Arizona, USA

**Keywords:** whole-genome sequencing, SNP clustering, transmission cluster, Thailand, *Mycobacterium tuberculosis*

## Abstract

Whole-genome sequence analysis is now widely used to delineate tuberculosis transmission clusters. A standard practice is to cluster bacterial isolates based on a fixed maximum genome-wide pairwise single nucleotide polymorphism (pwSNP) distance threshold. In this study, we evaluated the phylogenetic consistency of pwSNP-distance clustering with thresholds ranging between 1 and 25 single nucleotide polymorphisms (SNPs) using two contrasting data sets: (i) a data set from the UK (*N* = 390) published by T. M. Walker, C. L. C. Ip, R. H. Harrell, J. T. Evans, et al. (Lancet Infect Dis 13:137–146, 2013, https://doi.org/10.1016/S1473-3099(12)70277-3), which was foundational to the establishment of this method, and (ii) a data set from Thailand (*N* = 3,341), characterized by persistent transmission and sparse, non-systematic sampling. For the UK data set, the standard pwSNP-distance clustering using thresholds of ≥12 SNPs yielded entirely monophyletic clusters and showed high concordance with a comparative monophyly constrained, tree-based method. In contrast, for the Thai data set, pwSNP-distance clustering often generated non-monophyletic clusters, even by the 25-SNP threshold. The pwSNP-distance and comparative tree-based clustering methods only showed large consistency at thresholds of ≥22 SNPs. This suggests that SNP clusters defined by low distance thresholds (i.e., <12 SNPs for the UK data set, and <22 SNPs for the Thai data set) may lack robustness, and the problem is particularly severe for data sets characterized by persistent transmission, likely due to poorer cluster separation. Moreover, our findings indicate that large cluster sizes, high maximum intra-cluster genetic distances, and broad sample collection time spans may serve as useful indicators of potentially non-monophyletic clusters. We also demonstrate that mixed infections can produce spurious, phylogenetically long-range SNP linkages, underscoring the necessity of strict sequence quality control.

**IMPORTANCE:**

Fixed-threshold pairwise single nucleotide polymorphism (pwSNP)-distance clustering is commonly used to delineate tuberculosis transmission clusters. From an epidemiological perspective, a genuine transmission cluster must be monophyletic, originating from a single source. However, pwSNP-distance clustering is inherently simplistic and can therefore violate this principle, making the assessment of its phylogenetic consistency critical. Our results demonstrate that while this method effectively delineated complete transmission clusters for the data set from the UK, a low-burden and non-persistent transmission setting, it frequently generated non-monophyletic clusters when applied to the Thai data set, characterized by persistent transmission alongside sparse and non-systematic sampling. Furthermore, we found that clusters derived using low distance thresholds could notably vary between the pwSNP-distance and comparative tree-based clustering methods, suggesting limited reliability and robustness. To accurately delineate tuberculosis transmission clusters, especially for complex data from high-burden, endemic settings, we recommend transitioning from pwSNP-distance clustering toward more robust, phylogenetic clustering that respects evolutionary descent.

## INTRODUCTION

Despite continuous scientific and technological advances, tuberculosis (TB)—an airborne disease caused by bacteria within the *Mycobacterium tuberculosis* complex (MTBC)—remains a major global public health challenge. A fundamental component of effective TB control is a comprehensive understanding of its transmission dynamics; however, reconstructing complete TB transmission networks is intrinsically difficult. Traditional contact investigations rely heavily on patient interviews. However, TB transmission often occurs through casual and transient community contacts (1, 2), and subclinical/asymptomatic cases can contribute substantially to its spread (3–5). The clinical latency period of TB can also be very long and highly variable, ranging from months to years, or even a lifetime (6–9). Consequently, to gain a complete picture of how TB spreads, traditional contact investigation often requires patients to recall potential exposures that might be very distant in time, transient, and potentially unobvious. This is clearly very challenging, if not entirely impractical, and therefore frequently fails to link active cases to their original sources.

Whole-genome sequence (WGS) analysis provides a promising solution to these limitations by enabling TB transmission links to be inferred between cases lacking clear epidemiological connections based on the genetic similarity of their MTBC isolates. Conversely, it can also refute false epidemiological links assumed based on conventional molecular typing methods when isolates are clearly genetically distinct at the whole-genome level. These conventional typing methods include IS6110-based restriction fragment length polymorphism (IS6110-RFLP) analysis, which is based on the similarity of DNA fingerprints generated using IS6110 as a probe (10). In this method, bacterial isolates with identical IS6110 fingerprint patterns are inferred to be epidemiologically linked and assigned to the same transmission cluster. Other methods include spoligotyping, which examines variations within the direct repeat locus (11), now known to be the clustered regularly interspaced short palindromic repeats region of the bacteria (12), and mycobacterial interspersed repetitive unit (MIRU)-variable number tandem repeat (VNTR) typing, which assesses variations of VNTRs at 12–24 specific MIRU loci (13–15). Similar to the IS6110-RFLP analysis, isolates with the same spoligotypes or MIRU-VNTR patterns are clustered together to form transmission clusters. By analyzing the entire 4.4 million base pairs of the bacterial genome, WGS analysis offers unprecedented resolution over these conventional genotyping methods. Indeed, many studies have demonstrated that isolates showing identical genotypes as determined by these conventional methods can be highly divergent at the whole-genome level (16–19), thereby invalidating transmission links inferred by these lower-resolution approaches.

A common WGS analytical approach to delineating TB transmission clusters involves calculating pairwise single nucleotide polymorphism (pwSNP) distances between sample pairs and applying a fixed maximum distance threshold, commonly 12 single nucleotide polymorphisms (SNPs), to construct SNP-linked networks. This framework, hereafter referred to as fixed-threshold pwSNP-distance clustering, was first shown to be effective by Walker et al. (18) using MTBC WGS data from the UK, a low-burden and non-endemic setting. Since then, this method has been adopted to supplement TB national surveillance programs in many countries, such as the UK (20), the United States (21), and Australia (22, 23), with many others intending to follow suit. However, it is becoming increasingly evident that this method may not be universally applicable, particularly for settings defined by intermediate to high TB burden and/or persistent transmission (24, 25). Despite these emerging concerns, fixed-threshold pwSNP-distance clustering continues to be applied to WGS data from high-burden settings, often without adequate data set-specific validation (26–29).

In this study, we evaluated the phylogenetic consistency of the fixed-threshold pwSNP-distance clustering method using a range of thresholds (1–25 SNPs). We first validated our analysis protocol using the UK data set published by Walker et al. (18), which was foundational to the establishment of this method. We then applied it to a data set from Thailand, which is characterized by endemic, persistent transmission and sparse, non-systematic sampling. The generated clusters were mapped to their corresponding phylogeny to assess monophyly and were compared against clusters generated by an alternative, monophyly constrained, tree-based clustering approach, which was optimized to approximate the SNP clusters as closely as possible. Our findings reveal limitations of the method for inferring TB transmission clusters and highlight a lack of robustness in the results, especially when low distance thresholds are used to delineate clusters. This emphasizes the need for caution when applying it to complex data sets.

## MATERIALS AND METHODS

### Sequence data analysis protocol

Detailed information on the sequence data analysis protocol and program settings is provided in the supplemental Methods. Briefly, raw FASTQ files were validated using fqtools v2.0 (30). Adapter and low-quality base trimming were performed with Trimmomatic v0.40 (31). Read quality before and after cleaning was assessed with FastQC v0.12.1 (32) and summarized using MultiQC v1.33 (33).

Cleaned reads were then mapped to the *Mycobacterium tuberculosis* H37Rv reference genome (NC_000962.3) with BWA-MEM v0.7.19-r1273 (34), and duplicate reads were marked using Picard v3.4.0 (35) via GATK MarkDuplicates v4.6.2.0 (36–38). Read mapping statistics were computed using SAMtools coverage v1.23 (39, 40). Sample-wise variant calling with respect to the reference genome was performed using GATK HaplotypeCaller v4.6.2.0 (36–38) with ploidy set to two to enable detection of within-sample polymorphic sites. Polymorphic sites were extracted from the resulting gVCF files using BCFtools query v1.23 (39, 40), and counts were computed for each sample, considering only sites with a minor allele frequency (MAF) ≥0.05. Lineage assignment was performed using mtbtyper v1.0.0 (41) for all samples. For samples with a median read-mapping depth ≥5×, TBtypeR v25.1.0 (42) was used to detect samples with a strong multiple-strain signal (hereafter referred to as mixed samples).

Samples with an average read-mapping depth <10×, genome coverage <90%, >10,000 polymorphic sites, or a mean MAF > 0.4 were excluded from further analyses at this stage. The remaining gVCFs were filtered with BCFtools filter v1.23 (39, 40) to mask sites with a depth <10× and SNPs located within 10 bases of an indel. Joint genotyping was then performed using GATK GenomicsDBImport and GenotypeGVCFs v4.6.2.0 (36–38), and the resulting multi-sample VCF file was filtered to include only high-confidence biallelic SNPs with GATK SelectVariants and VariantFiltration v4.6.2.0 (36–38). Sites located within refined low-confidence or low pileup mappability regions as defined in reference 43, as well as genes associated with known drug resistance, were also masked using BCFtools view v1.23 (39, 40). This was done to minimize potential mapping artifacts and false clustering signals arising from convergent evolution.

A multiple sequence alignment (MSA) was subsequently constructed from the resulting multi-sample VCF file, ignoring masked sites. Polymorphic sites were genotyped using IUPAC ambiguous nucleotide codes, and uncalled or missing sites were genotyped as “*N.*” Columns with >80% missing or ambiguous bases were removed, and samples with >20% remaining missing or ambiguous bases were further excluded from downstream analyses. Fully conserved invariant sites (ignoring the remaining missing or ambiguous bases) were also removed from the MSA to generate a SNP-only alignment.

A maximum likelihood (ML) phylogeny was then reconstructed from the clean MSA with IQ-TREE v3.0.1 (44). The best-fit nucleotide substitution model was automatically selected by ModelFinder (45), implemented within the program under the default Bayesian information criterion, and used in the reconstruction. Base ascertainment bias was corrected by manually providing counts of invariant nucleotide sites directly to the program. In parallel, pwSNP distances were calculated from the clean MSA using snp-dists v1.2.0 (46) with default settings.

Samples were considered “SNP-linked” if their pwSNP distance fell at or below a defined threshold. “SNP clusters” were defined as the connected components of an undirected graph in which edges existed only between SNP-linked samples.

### Data sets analyzed in this study

#### Validation data set—Walker et al., 2013

The *Mycobacterium tuberculosis* data set published by Walker et al. (18) was used to validate our sequence data analysis protocol (see below). The data set comprised 390 samples categorized into four overlapping sub-data sets (detailed in supplemental file S2 of the original publication):

- C-1, consisting of 49 pairs of cross-sectional pulmonary and extrapulmonary samples obtained within one month of each other (2 samples per patient, *N* = 98);
- C-2, consisting of 30 longitudinal pulmonary collections separated by at least 6 months (2–9 samples per patient, *N* = 110);
- C-3, consisting of 25 household collections (2–19 samples per household, *N* = 86); and
- C-4, consisting of 11 MIRU-VNTR-defined community cluster collections (6–54 samples per cluster, *N* = 217). For this sub-data set, epidemiological information was available for 158 patient pairs, graded as having a “known link” (*N* = 68 pairs), “possible link” (*N* = 13 pairs), or “no known link” (*N* = 77 pairs; detailed in supplemental file S6 of the original publication).

Sequence data of all 390 samples passed our quality control procedures.

#### Thai data set

The primary data set evaluated in this study comprised 4,594 MTBC samples collected in Thailand. All sequence data were publicly available. Using the sequence data analysis protocol described above, 3,341 samples passed quality control and were retained for downstream analysis.

### Validation of our analysis protocol by reproducing Walker et al. (2013) results

To validate our sequence data analysis protocol, we constructed pwSNP distance distributions for the four Walker et al. (18) sub-data sets that were originally used to propose the 5- and 12-SNP thresholds for epidemiological linkage inference. Paralleling the original study’s methodology, distributions for C-1 and C-2 were constructed using maximum intra-host pwSNP distances. For C-3, we constructed a minimum spanning tree (MST) for each household, using the minimum pwSNP distances between all unique patient pairs as the starting point, and derived the distribution from the resulting MSTs. For C-4, separate distributions were constructed for the three epidemiological linkage grades using the minimum pwSNP distances between patient pairs within each MIRU-VNTR community cluster. These resulting distributions were then evaluated to identify suitable pwSNP distance thresholds for epidemiological inference and compared with the original findings.

### Global pwSNP distance distributions and overall phylogenetic concordance of inferred SNP linkages

We also analyzed the distributions of pwSNP distances derived from both a fully connected pairwise graph and a global MST to identify more broadly suitable pwSNP thresholds for clustering. Inferred SNP linkages were mapped onto the estimated phylogeny to assess their general phylogenetic concordance. Additionally, we calculated the cophenetic correlation coefficient, i.e., the Pearson correlation between the pwSNP distances and the tree-derived cophenetic distances, to quantify the broad relationship between the two metrics.

### Conversion of pwSNP distances to time intervals

Converting pwSNP distances to time intervals requires estimates of evolutionary rates. To this end, we conducted root-to-tip regression analyses under the molecular clock assumption, excluding mixed samples and samples with high proportions of ambiguous bases. The slope of the fitted linear model (i.e., evolutionary rate estimate) was calculated for each lineage individually, alongside a two-sided 95% confidence interval (CI), and a one-sided *P*-value to test for a significantly positive slope. Both calculations used the Šidák adjustment to correct for multiple comparisons. Significant molecular clocks were then used to convert pwSNP distances to time intervals.

### Comparative monophyly constrained tree-based clustering

To generate monophyly constrained tree-based clusters that were most consistent with the SNP clusters, we first identified the cophenetic distance threshold optimized for predicting the pwSNP clustering results. This was done with receiver operating characteristic (ROC) analysis, using the binary status of sample pairs (i.e., whether they belonged to the same SNP cluster or not) as the response variable and their cophenetic distance as the predictor. Overall predictive performance was quantified using the area under the curve (AUC). The optimal cophenetic distance was identified using the “closest top-left” method, determined by minimizing the quantity ${\left(1-sensitivity\right)}^{2}+{\left(1-specificity\right)}^{2}$. Monophyly constrained tree-based clusters were then generated using this optimal cophenetic threshold, defined as the largest possible monophyletic clades within which no internal pairwise cophenetic distance exceeded the threshold.

### Evaluation of overall phylogenetic consistency of fixed-threshold pwSNP-distance clustering

To evaluate the overall phylogenetic consistency of the fixed-threshold pwSNP-distance clustering method across thresholds ranging from 1 to 25 SNPs, we used the estimated ML phylogeny as the reference background and computed and analyzed the following for each threshold:

- The odds of a sample being assigned to a multi-sample cluster versus remaining a singleton, calculated via binomial logistic regression using the log-transformed SNP threshold as the predictor;
- The total number of multi-sample clusters; and
- The strict monophyly frequency of the multi-sample SNP clusters.

Furthermore, the overall concordance between the pwSNP- and tree-based clustering approaches was quantified using three metrics: the adjusted Rand index (ARI), normalized mutual information (NMI), and variation of information (VI). Mixed samples and samples with high proportions of ambiguous bases were excluded from these analyses.

### In-depth phylogenetic classification and characterization of multi-sample SNP clusters estimated for the Thai data set using 5-, 12-, and 22-SNP thresholds

For each multi-sample SNP cluster, we first identified the most recent common ancestor (MRCA) of its members to define its “base clade.” We then identified tree-based clusters that shared at least one member with the SNP cluster, and defined an “expanded clade” based on the MRCA of all these members combined. We also mapped the cluster membership onto the global phylogeny as a binary trait and conducted maximum parsimony ancestral state reconstruction to estimate the cluster membership at all internal nodes. SNP clusters were classified as monophyletic, paraphyletic, polyphyletic, or para/polyphyletic based on the maximum parsimony ancestral cluster membership state at the MRCA node of the base clade. Monophyletic SNP clusters were further classified using their overlapping tree-based clusters as a comparative reference: “optimally monophyletic” (perfectly matching the overlapping tree-based cluster), “underclustering monophyletic” (monophyletic but smaller than its overlapping tree-based cluster), or “overclustering monophyletic” (monophyletic but containing multiple tree-based clusters). Shifts in the distribution of these phylogenetic classes across evaluated SNP thresholds were assessed using multinomial logistic regression.

To further characterize the SNP clusters, we computed their maximum intra-cluster pwSNP distance, maximum intra-cluster cophenetic distance, and the overall temporal span of sample collection. We also computed SNP cluster purity (the ratio of the SNP cluster size to the total number of tips within its base clade), the number of “intruders” (non-SNP cluster members within the base clade), and the number of “outruders” (samples outside the base clade but within the expanded clade). Additionally, we evaluated the homoplasy of the inferred ancestral cluster assignments to calculate the Fitch parsimony score, consistency index, and retention index for each SNP cluster.

Next, we performed a Kruskal-Wallis test to compare the distributions of these properties across the different phylogenetic classes. All properties showed significant variation across classes (*P* < 0.05). Consequently, we performed *post-hoc* pairwise comparisons using Dunn’s test for all properties, applying the Benjamini-Hochberg procedure to control the false discovery rate. The comparison results were summarized using a compact letter display to denote statistical groupings, where classes sharing at least one common letter are not significantly different at an alpha level of 0.05.

## RESULTS

### Validation of our sequence data analysis protocol by reproducing Walker et al. (2013) results

**Fig 1:**
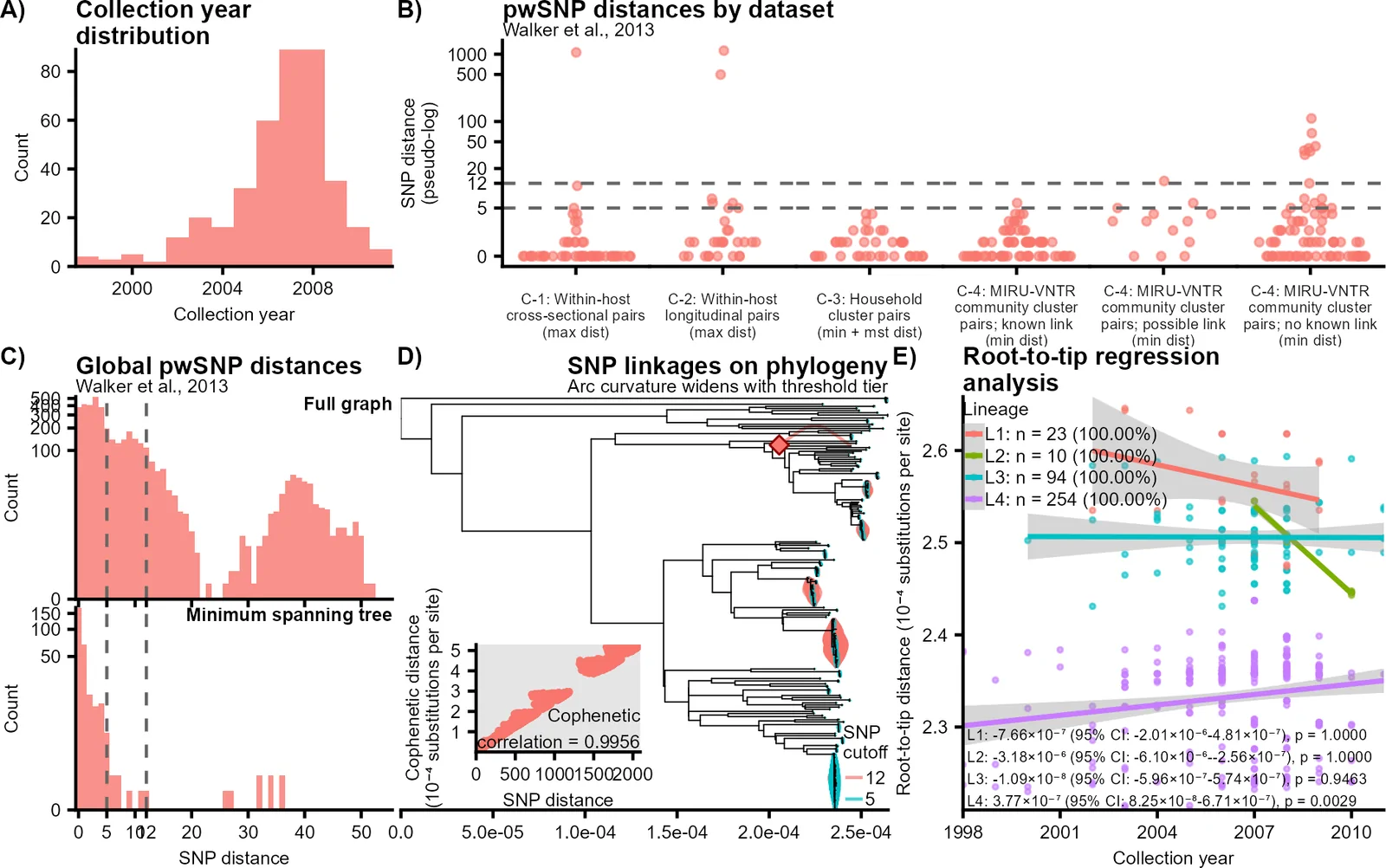
Validation of our sequence data analysis protocol by reproducing results published by Walker et al. (18). (**A**) Collection year distribution of samples passing quality control (390/390 = 100%). (**B**) pwSNP distances of the four Walker et al. (18) sub-data sets (C-1 to C-4, with C-4 subdivided into three epidemiological linkage grades: “known link,” “possible link,” and “no known link”). Dashed lines indicate the established 5-SNP and 12-SNP thresholds. The *y*-axis is pseudo-log transformed to accommodate large outlier values. (**C**) Distribution of pwSNP distances derived from the global complete, fully connected pairwise network graph of all 390 samples (top), and from the global minimum spanning tree (bottom). The fully connected network graph was derived from a global pwSNP distance matrix computed from a clean MSA of 16,876 sites, generated by our sequence data analysis protocol. Dashed lines denote the 5- and 12-SNP thresholds. The *x*-axis of both histograms is capped between 0 and 55 SNPs. (**D**) Mid-point rooted ML phylogenetic tree of all 390 samples with inferred SNP linkages overlaid. The tree was constructed with the following constant site distribution: A, 706,719; C, 1,337,491; G, 1,332,000; and T, 707,913, and used the TVM + *F* + *I* nucleotide substitution model, which was determined as the best-fit model under the Bayesian information criterion. Arcs indicate linkages supported by ≤5 SNPs (blue) and 6–12 SNPs (red), with arc curvature widened by threshold tier. One mixed sample was detected, indicated by the diamond symbol. (Inset) Correlation between pwSNP distances and phylogenetic cophenetic distances. (**E**) Root-to-tip regression analysis to evaluate the molecular clock signal across four main lineages (L1–L4), excluding the mixed sample. Solid lines and shaded regions represent linear regression fits and their 95% confidence intervals, respectively. Inset text shows the estimated slope (molecular clock rate, substitutions per site per year) for each lineage, along with the corresponding two-sided 95% confidence interval and one-sided *P*-value for testing a positive slope, both adjusted for multiple comparisons using the Šidák adjustment method.

We validated our sequence data analysis protocol by applying it to the data set published by Walker et al. (18), which was originally used to propose the 5- and 12-SNP thresholds as suitable for epidemiological linkage inference and for pwSNP-distance clustering analysis. The data set comprised 390 samples collected between 1998 and 2011 (Fig. 1A) (Table S1; L1, *N* = 23; L2, *N* = 10; L3, *N* = 94; L4, *N* = 254; L5, *N* = 2; *Mycobacterium bovis*, *N* = 6; mixed sample, *N* = 1) and were categorized into four overlapping sub-data sets (C-1 to C-4; see Materials and Methods). Our analysis generated pwSNP distance distributions that were highly consistent with the original study’s finding—sample pairs from patients with known direct epidemiological linkages generally differed by five or fewer SNPs, whereas such information was typically absent for pairs differing by more than 12 SNPs (Fig. 1B). This confirms the validity of the previous findings and also our sequence data analysis protocol.

To evaluate the SNP clustering thresholds more broadly (i.e., independent of known epidemiological linkages, which are commonly unavailable or largely incomplete in many practical settings), we analyzed the pwSNP distance distributions derived from both the fully connected pairwise network and the MST graph of all 390 samples. Although the pwSNP distance distribution derived from the fully connected pairwise network did not support either the 5- and 12-SNP threshold as a suitable clustering cutoff (Fig. 1C, top), the distribution derived from the MST clearly supported the 12-SNP threshold (Fig. 1C, bottom). Overlaying SNP linkages inferred using the 5- and 12-SNP thresholds onto the phylogeny did not show any clear phylogenetic discordance (Fig. 1D), and the overall concordance between pwSNP and cophenetic distances was high (cophenetic correlation = 0.9956; Fig. 1D, inset plot). However, we noted one 12-SNP linkage connecting a pair of apparently phylogenetically distant samples. Examination revealed that this linkage involved a mixed sample (indicated by the diamond symbol in Fig. 1D), which also exhibited the highest rate of ambiguous bases in the alignment (1.25%) and was placed much deeper in the tree relative to its linked sample.

Next, assuming the molecular clock, we estimated evolutionary rates from the data and used them to compute equivalent temporal separation distances for the 5- and 12-SNP thresholds. The clock rates were computed separately for the four main lineages (L1–L4, *N* ≥ 10) using root-to-tip regression analysis, excluding the detected mixed sample (Fig. 1E). Our analysis suggested that the clock signal differed significantly among lineages (analysis of variance [ANOVA] test: df = 3; *F* = 5.05; *P*-value = 1.94 × 10^−3^), and only L4 showed a significantly positive clock rate (Fig. 1E, inset text), estimated at 3.77 × 10^−7^ (95% CI: 8.25 × 10^−8^ to 6.71 × 10^−7^) substitutions/site/year × (16,876 variant sites in the MSA + 4,084,123 invariant sites) = 1.54 (95% CI: 0.34–2.75) SNPs/genome/year. Based on this rate, 5 and 12 pwSNPs correspond to total temporal separations of 3.24 (95% CI: 1.82–14.78) years and 7.77 (95% CI: 4.36–35.47) years, respectively.

### pwSNP-distance clustering with a threshold of 12 SNPs or greater gave phylogenetically consistent results for the Walker et al. (2013) data set

**Fig 2:**
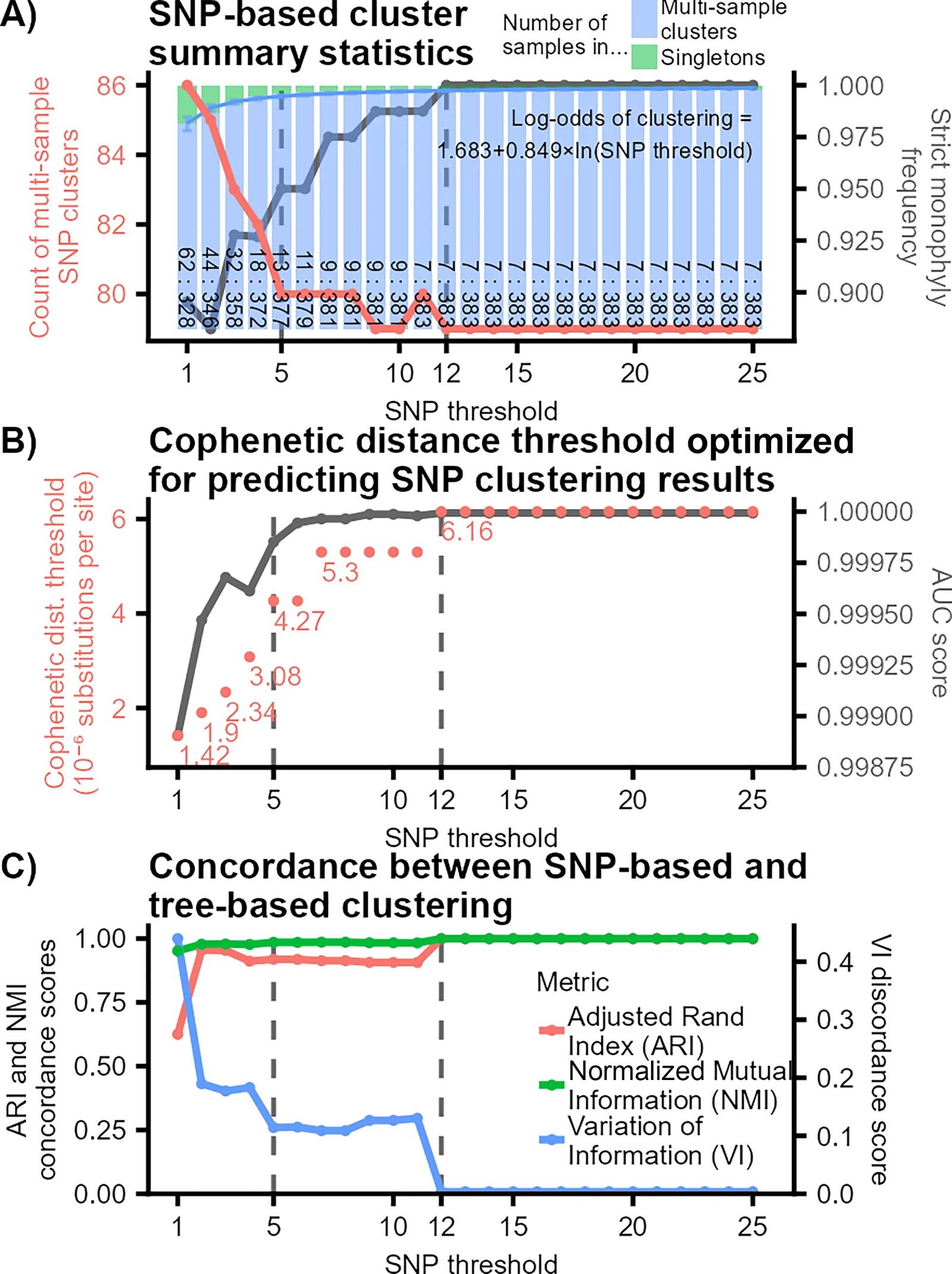
Phylogenetic consistency analysis of pwSNP-based clustering applied to the Walker et al. data set (18). (**A**) Count of multi-sample SNP clusters (left primary *y*-axis; red line), frequency of monophyletic clusters among multi-sample SNP clusters (right secondary *y*-axis; gray line), and proportion of samples assigned to singletons vs. multi-sample clusters (stacked bar charts; green-top and blue-bottom, respectively; absolute ratios are shown at the bottom) derived using pwSNP clustering analysis with SNP thresholds ranging from 1 to 25 SNPs. The solid blue line and error bars depicted across the stacked bar charts represent the predicted proportions and confidence intervals derived from a binomial logistic regression model (equation shown top right). (**B**) Cophenetic distance thresholds best predicting the pwSNP-distance clustering results at each SNP threshold, identified using ROC analysis (left primary *y*-axis; red points; values shown if they change from the immediately preceding value). AUC scores are also shown to indicate the overall predictive performance of the optimized cophenetic distance (right secondary *y*-axis; gray line). (**C**) Consistency between pwSNP-based and tree-based clustering. Concordance is measured using the adjusted Rand index (ARI; red line), and normalized mutual information (NMI, green line; primary *y*-axis), and discordance is measured using the variation of information (VI; blue line; secondary *y*-axis). Vertical dashed lines in all plots indicate the 5- and 12-SNP thresholds.

We then systematically evaluated the overall phylogenetic consistency of the fixed-threshold pwSNP-distance clustering method across thresholds ranging from 1 to 25 SNPs. Logistic regression analysis revealed that increasing the SNP threshold significantly shifted the distribution of sample assignments from singletons to multi-sample clusters (analysis of deviance: deviance = 215.57, df = 1, *P*-value < 0.0001). The shift was well approximated by a log-logit model; for every doubling of the SNP threshold, the odds of a sample being assigned to a multi-sample cluster were estimated to increase by a factor of 1.801 (Fig. 2A, stacked bars). Concurrently, increasing the threshold generally led to a decrease in the total number of multi-sample SNP clusters, indicative of cluster merging (Fig. 2A, left axis), and an increase in the frequency of monophyletic clusters among multi-sample SNP clusters (Fig. 2A, right axis). Both values stabilized at and beyond the 12-SNP threshold (multi-sample clusters = 79; monophyletic frequency = 1.00).

To further evaluate the overall internal phylogenetic consistency of these SNP clusters, we used ROC analysis to determine the optimal cophenetic distance that best predicted the pwSNP clustering results at each threshold (Fig. 2B). We then used them to derive monophyly constrained tree-based clusters and compared the results from the two clustering approaches (see Materials and Methods; see Table S2 for pwSNP-based and tree-based clustering results). We found that, at thresholds of 12 SNPs or greater, the results from pwSNP-based clustering and tree-based clustering showed near-absolute concordance as measured by the ARI (0.9999), NMI (0.9995), and VI (0.0036; Fig. 2C). Figure S1 shows a two-dimensional *t*-SNE projection of the cophenetic distances, visually illustrating the concordance between the pwSNP-based and tree-based clusters at the 12-SNP threshold. The resulting clusters derived using the threshold of five SNPs were also shown for comparison. Collectively, these results showed that pwSNP-distance clustering with a fixed threshold of 12 SNPs or greater yielded phylogenetically consistent and robust results for this data set.

### Analysis of the Thai data set

**Fig 3:**
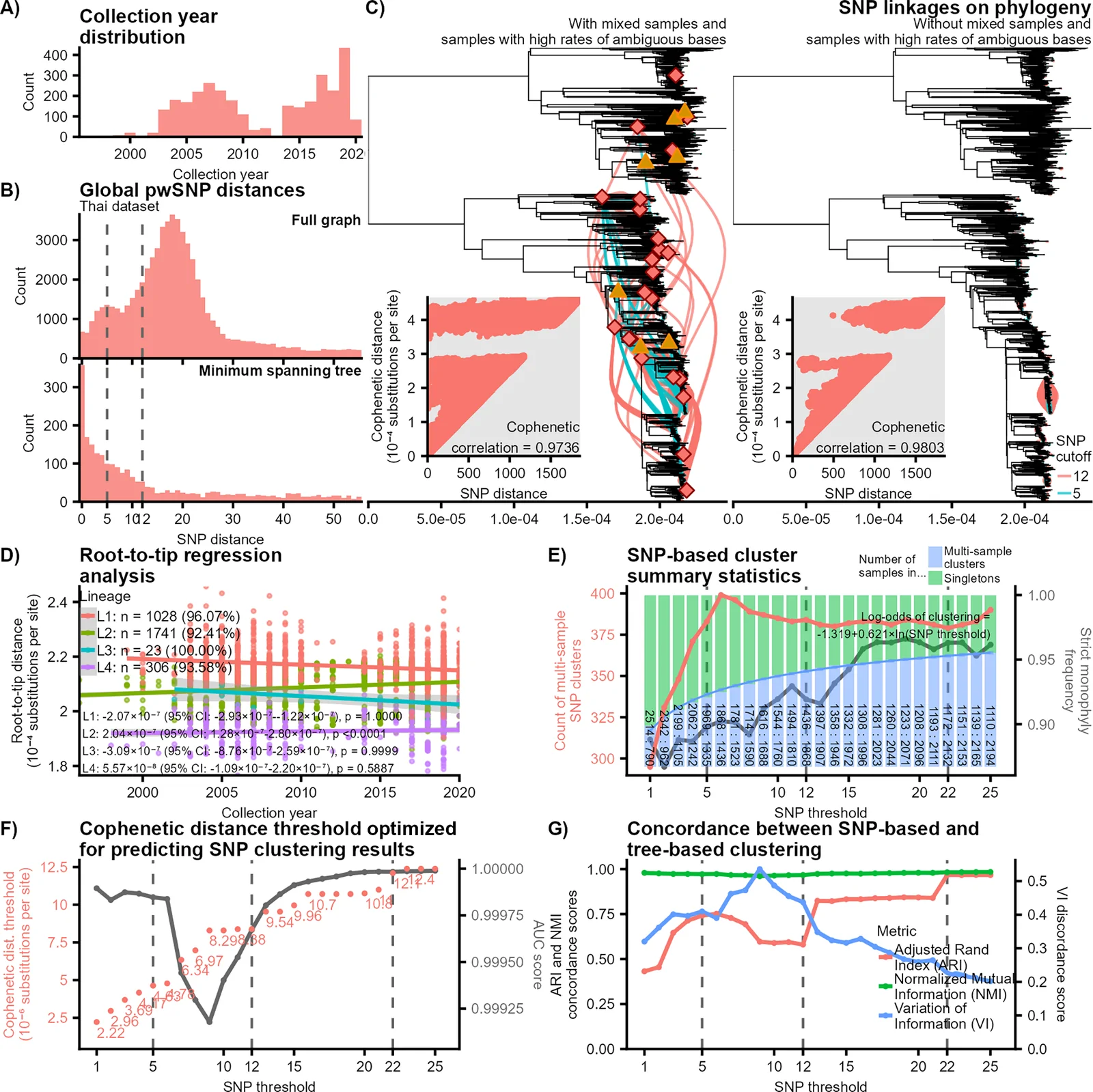
Analysis of the Thai data set comprising 3,341 samples passing quality control. (**A**) Collection year distribution of samples passing the quality control. (**B**) Global pwSNP distance distributions inferred from a fully connected pairwise network of all samples (top), and from the global minimum spanning tree (bottom). The fully connected pairwise network was derived from a pwSNP distance matrix computed from a clean MSA of 116,846 sites. The *x*-axis of both histograms is capped between 0 and 55 SNPs. (**C**) ML phylogenies with inferred SNP linkages overlaid, rooted with three *M. bovis* samples (not shown). The tree was constructed with the following constant site distribution: A, 695,281; C, 1,298,953; G, 1,293,599; and T, 696,459, and the TVM + *F* + R7 nucleotide substitution model was used in the reconstruction, determined as the best-fit model under the Bayesian information criterion. The left panel includes 27 mixed samples (highlighted by red diamond symbols) and 7 samples with high proportions of ambiguous bases in the alignment (>0.1%, orange triangle symbols), while the right panel excludes them. (**D**) Root-to-tip regression analysis to evaluate the molecular clock signal across four main lineages (L1–L4), excluding the mixed samples and samples with high rates of ambiguous bases. Data are presented and formatted as described in Fig. 1A, C and D. (**E**) SNP cluster statistics, (**F**) optimal cophenetic distance thresholds, and (**G**) consistency between pwSNP-based and tree-based clustering results evaluated for SNP thresholds ranging from 1 to 25 SNPs. Data are presented and formatted as described in Fig. 2A through C. Vertical dashed lines in all plots indicate the 5- and 12-SNP thresholds, with the addition of the 22-SNP threshold, from which the pwSNP-based and tree-based clustering started to yield largely consistent results.

After validating our sequence data analysis protocol and demonstrating the phylogenetic consistency of the standard pwSNP-distance clustering using the Walker et al. (18) data set, we applied the same analyses to the Thai data set. The data set comprised 4,594 samples, 3,341 of which passed quality control (Table S3; L1, *N* = 1,074; L2, *N* = 1,887; L3, *N* = 23; L4, *N* = 327; *M. bovis*, *N* = 3; mixed samples, *N* = 27). The data were obtained from multiple publicly accessible databases, generated by multiple sequencing projects. As evident from the distribution of collection years (1996–2020), sampling was not systematic, with notably sparse sampling between 2011 and 2013 (Fig. 3A). The distributions of pwSNP distances derived from the fully connected pairwise network (Fig. 3B, top) and the global MST (Fig. 3B, bottom) did not support either the 5- and 12-SNP threshold as a robust SNP clustering cutoff. In fact, both distributions were entirely continuous, lacking any clear gaps between 0 and 55 SNPs that would be suitable for placing robust SNP distance cutoffs for clustering.

Overlaying the 5- and 12-SNP linkages onto the estimated phylogeny initially showed numerous phylogenetically long-range SNP linkages (Fig. 3C, left), as well as many sample pairs with high cophenetic distances but relatively low pwSNP distances (Fig. 3C, left inset; *r* = 0.9736). Manual inspection revealed that most of these phylogenetically long-range SNP linkages involved mixed samples, again exhibiting comparatively high ambiguous base percentages—26 of the top 27 samples with the highest rates of ambiguous bases in the alignment were mixed samples, showing rates ranging between 0.182% and 1.469%; the one remaining detected mixed sample had an ambiguous-base rate of 0.063% (Table S3). After removing the 27 mixed samples and 7 samples having >0.1% ambiguous bases in the alignment (including 4 L1 samples and 3 L2 samples), these phylogenetically long-range SNP linkages were no longer apparent (Fig. 3C, right). Moreover, the majority of pwSNP/cophenetic distance discordant sample pairs also disappeared, resulting in an improved cophenetic correlation coefficient (Fig. 3C, right inset; *r* = 0.9803).

Similar to the results yielded from the analysis of the Walker et al. (18) data set, a root-to-tip regression excluding the detected mixed samples and those with high rates of ambiguous bases (Fig. 3D) suggested that the molecular clock signal differed significantly among lineages (ANOVA test: df = 3; *F* = 27.541; *P*-value < 0.0001). However, for the Thai data set, only L2 showed a significantly positive clock rate (Fig. 3D, inset text), estimated at 2.04 × 10^−7^ (95% CI: 1.28 × 10^−7^ to 2.80 × 10^−7^) substitutions/site/year × (116,846 variant sites in the MSA + 3,984,292 invariant sites) = 0.84 (95% CI: 0.53–1.15) SNPs/genome/year.

We subsequently investigated the overall phylogenetic consistency of the fixed-threshold pwSNP-distance clustering method using SNP thresholds ranging between 1 and 25 SNPs. Again, increasing the SNP threshold significantly shifted the distribution of samples from singletons into multi-sample clusters (analysis of deviance: deviance = 4856.2, df = 1, *P*-value < 0.0001), and the shift could be well approximated by a log-logit model (Fig. 3E). The odds of a sample being assigned to a multi-sample cluster were estimated to increase by a factor of 1.538 for every doubling of the SNP threshold. Also, unlike the Walker et al. (18) data set, neither the total number of multi-sample clusters nor the monophyly frequency stabilized, even up to the 25-SNP threshold (Fig. 3E).

Furthermore, for each SNP threshold, the optimal cophenetic distance for predicting sample pairs belonging to the same SNP clusters was computed (Fig. 3F) and used to derive monophyly constrained tree-based clusters (Table S4). We found that the optimal cophenetic distance threshold itself showed no clear sign of stabilizing, even up to the 25-SNP threshold (Fig. 3F). The ARI and NMI concordance scores suggested that pwSNP-based and tree-based clustering only became largely consistent at SNP thresholds of 22 or greater (Fig. 3G, left axis). Nonetheless, the VI discordance score indicated that inconsistencies still existed even at these high SNP clustering thresholds (Fig. 3G, right axis). Figure S2 shows a two-dimensional *t*-SNE projection of the cophenetic distances, showing the varying degrees of overlap and discrepancy between the pwSNP-based and tree-based clusters at the 5-, 12-, and 22-SNP thresholds.

Overall, our results demonstrated that the 5- and 12-SNP thresholds were unsuitable for this data set from Thailand. To derive clusters that were largely consistent with the comparative monophyly constrained tree-based clustering, a high SNP threshold of at least 22 SNPs was required. Given the estimated clock rate of L2, the only lineage with a significant rate of 0.84 (95% CI: 0.53–1.15) SNPs/genome/year, a 22-pwSNP difference is equivalent to an estimated total temporal separation of 26.29 (95% CI: 19.16–41.86) years.

### Further phylogenetic classification and properties of multi-sample SNP clusters inferred for the Thai data set using 5-, 12-, and 22-SNP thresholds

To evaluate the phylogenetic properties of the multi-sample SNP clusters inferred for the Thai data set, we examined those generated at the 5-, 12-, and 22-SNP thresholds. Using the estimated ML phylogeny and monophyly constrained tree-based clusters as a reference background, they were classified into six phylogenetic classes: (i) optimally clustering monophyletic (perfectly matching the overlapping tree-based cluster), (ii) underclustering monophyletic (monophyletic, but smaller than its overlapping tree-based cluster), (iii) overclustering monophyletic (monophyletic, but containing multiple tree-based clusters), (iv) paraphyletic, (v) polyphyletic, and (vi) para/polyphyletic (maximum parsimony ancestral state reconstruction could not definitively determine the cluster assignment at the clade’s MRCA).

**Fig 4:**
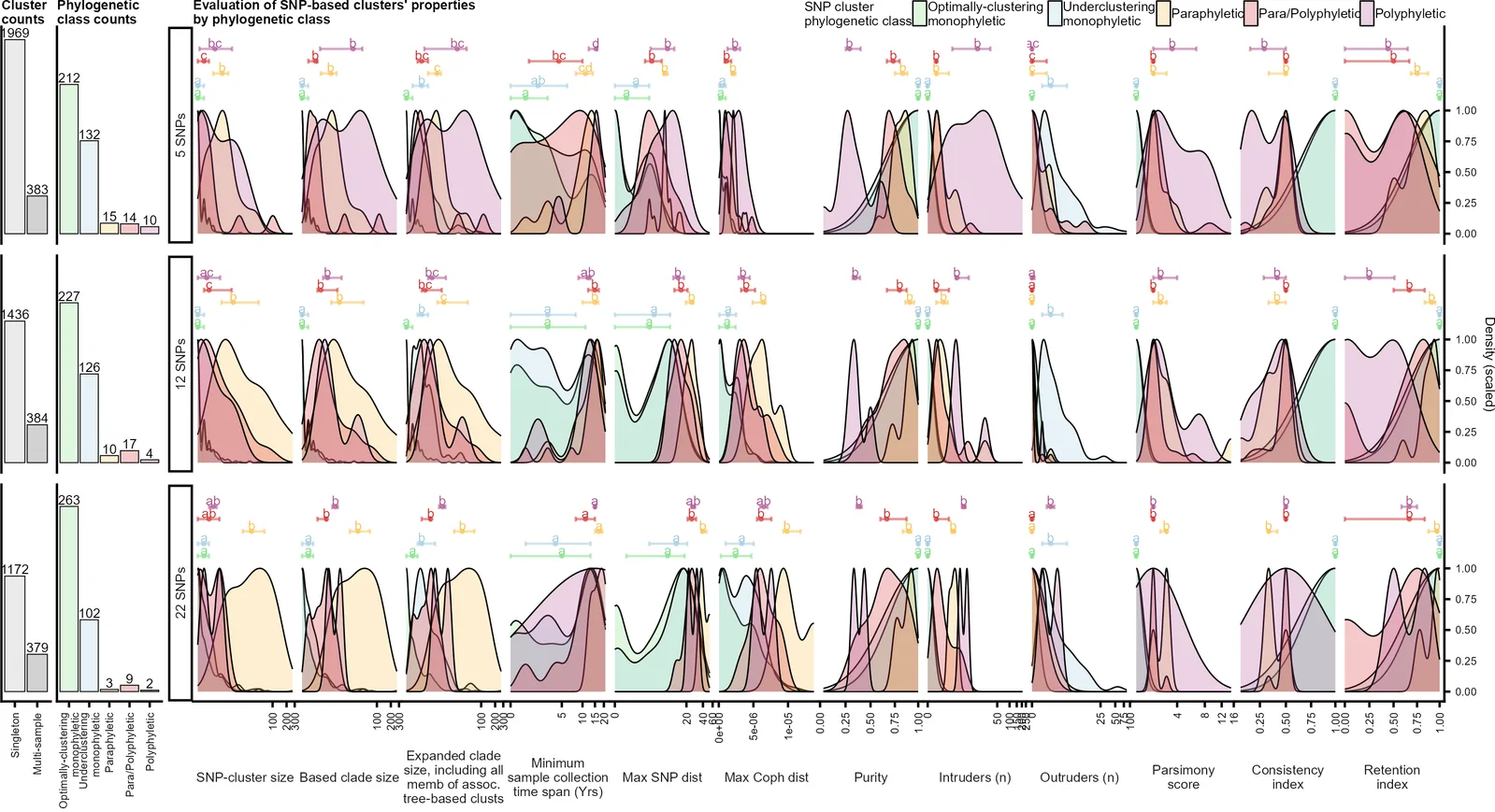
Phylogenetic classification and properties of multi-sample SNP clusters derived for the Thai data set using 5-, 12-, and 22-SNP clustering thresholds. (Left) Bar plots showing counts of singletons versus multi-sample SNP clusters at the three clustering thresholds. (Middle) Bar plots showing counts of multi-sample SNP clusters assigned to each phylogenetic class at the respective thresholds. (Right) Scaled density distributions of 12 cluster properties (columns) grouped by the evaluated SNP threshold (rows). Horizontal bars above the density plots represent the interquartile range (Q1–Q3), with a central point indicating the median. Letters above the horizontal bars denote statistical groupings derived from Dunn’s *post-hoc* pairwise comparisons (detailed in Tables S5 and S6) and are summarized via a compact letter display; classes sharing at least one common letter do not differ significantly at an alpha level of 0.05. The *x*-axes are pseudo-log transformed to accommodate wide data ranges.

Across all three thresholds, optimally clustering monophyletic clusters were the most common, followed by underclustering monophyletic, para/polyphyletic or paraphyletic, and polyphyletic clusters, respectively; no overclustering monophyletic clusters were observed (Fig. 4; see Fig. S3 and S4 for phylogenetic visualizations of all non-optimal clusters). Multinomial logistic regression suggested that changing the SNP threshold significantly affected the distribution of phylogenetic classes (analysis of deviance: deviance = 25.672, df = 4, *P*-value < 0.0001). Specifically, the analysis revealed a significant negative relationship between the SNP threshold and the log-odds of a cluster being classified as underclustering monophyletic (coefficient = −0.0284, SE = 0.0094), paraphyletic (coefficient = −0.1012, SE = 0.0334), polyphyletic (coefficient = −0.1154, SE = 0.0461), or para/polyphyletic (coefficient = −0.0385, SE = 0.0241), relative to the optimally clustering monophyletic baseline. This means that, as the SNP threshold increases, a greater proportion of optimally clustering monophyletic clusters is generated.

Comparisons of cluster properties revealed significant variation across the phylogenetic classes for all investigated metrics at all evaluated thresholds (Kruskal-Wallis test, maximum *P*-value = 0.0142; Table S5). *Post-hoc* Dunn’s test further revealed that optimally and under-clustering monophyletic clusters had highly similar property profiles (Fig. 4). As expected by definition, the main distinctions between the two were that the latter had a larger expanded clade size, including all members of corresponding tree-based clusters, and the presence of “outruders,” which occur when pwSNP-based clustering fails to capture the broader clade defined by the comparative tree-based cluster. Paraphyletic, polyphyletic, and para/polyphyletic clusters also shared similar property profiles among themselves. Overall, compared to monophyletic clusters, they were generally associated with larger phylogenetic clades, greater maximum intra-cluster genetic distances, and broader temporal spans of sample collection. Notably, across all three evaluated thresholds, these non-monophyletic classes showed statistically indistinguishable distributions for purity scores, the numbers of non-member “intruders” within their clades, and phylogenetic homoplasy (measured by parsimony score, consistency index, and retention index), but all of which deviated significantly from those of the monophyletic clusters. These results suggest a robust and stable statistical demarcation between monophyletic and problematic non-monophyletic clusters.

## DISCUSSION

WGS analysis enables the inference of TB transmission clusters at a resolution unattainable by traditional contact investigations or conventional molecular typing methods, such as IS6110-RFLP, spoligotyping, and MIRU-VNTR. A widely adopted method for inferring TB transmission clusters from WGS data is to group samples using some predefined maximum genome-wide pwSNP distance thresholds. In this study, we refer to this method as fixed-threshold pwSNP-distance clustering.

Fundamentally, epidemiological principles dictate that a transmission cluster must originate from a single common ancestor and thereby form a monophyletic clade. However, calculation of pwSNP distances is inherently simplistic; it does not account for complex evolutionary phenomena, such as superimposed substitutions, unequal equilibrium base frequencies, variable substitution rates among different pairs of nucleotide bases, or variable evolutionary rates among lineages or genomic positions. Consequently, pwSNP-based clustering can violate the principle of evolutionary descent, making assessment of its phylogenetic consistency critical. Despite its widespread use, studies evaluating the phylogenetic consistency of this method remain limited. To address this gap, in this study, we evaluated the phylogenetic consistency of the fixed-threshold pwSNP-distance clustering method using data sets from two contrasting epidemiologic settings: (i) a data set from the UK—a low-burden setting with limited transmission—published by Walker et al. (18), which is foundational to the establishment of this method, and (ii) a data set from Thailand—a high-burden, endemic setting, characterized by non-systematic data sampling.

Applying our sequence data analysis protocol to the Walker et al. (18) data set from the UK successfully reproduced the original findings—identifying thresholds of ≤5 and >12 SNPs as suitable cutoffs for inferring epidemiologically linked and unlinked sample pairs, respectively (Fig. 1B)—thereby validating our methodology. Furthermore, the distribution of pwSNP distances derived from the global MST revealed a natural break in the genetic continuum between 13 and 25 SNPs, upon which a reliable SNP threshold could be defined (Fig. 1C). Applying thresholds of ≥12 SNPs (equivalent to ≥7.77 [95% CI: 4.36–35.47] years of total evolutionary separation) yielded perfectly monophyletic clusters (Fig. 2A, gray line). This supports that fixed-threshold pwSNP-distance clustering can effectively delineate complete TB transmission clusters in a low-burden, non-persistent setting, where transmission chains terminate shortly after their emergence. In such settings, transmission clusters would manifest themselves in the phylogeny as well-defined “tippy” clades separated by long internal stem branches (Fig. 1D), creating a distinct microevolutionary structure that pwSNP-distance clustering can readily capture. Nevertheless, it is noteworthy that, even in this data set, lower SNP thresholds still yielded non-monophyletic multi-sample clusters (Fig. 2A, gray line), revealing the inherent limitations of this method for delineating transmission clusters of cases that are exclusively epidemiologically very closely linked.

Our results, however, indicate that the 5- and 12-SNP thresholds cannot be generalized to the Thai data set, likely due to fundamental differences in TB epidemiological dynamics. In fact, the pwSNP distance distribution derived from the global MST contained no discernible gaps between 0 and 55 SNPs (Fig. 3B), preventing the establishment of a single, universally applicable, and robust clustering threshold for this data set. This continuous distribution is a hallmark signature of persistent transmission chains characteristic of high-burden, endemic settings such as Thailand. Indeed, rather than showing well-defined tippy clades, the phylogeny estimated for the Thai data set showed a highly complex branching pattern toward the tips with highly variable internal branch lengths (Fig. 3C), explaining the observed continuous distribution. Consequently, the application of pwSNP-distance clustering to this data set produced results that failed to stabilize even at the 25-SNP threshold, and in fact, none of the evaluated thresholds produced entirely monophyletic clusters (Fig. 3E, gray line). Paralleling the observations made for the Walker et al. (18) data set, using lower thresholds produced non-monophyletic clusters more frequently than higher thresholds (Fig. 3E, gray line). This further supports that fixed-threshold pwSNP-distance clustering is unreliable for delineating tightly linked TB cases into transmission clusters.

To further evaluate the limitations of fixed-threshold pwSNP-distance clustering, we compared its clustering results with those obtained from a monophyly constrained, tree-based clustering method. In the absence of an epidemiological ground truth, we optimized the tree-based method to yield clusters that approximated the pwSNP clusters as closely as possible, providing a benchmark against which the robustness of the pwSNP clusters could be assessed. For the Walker et al. (18) data set, SNP clusters derived using thresholds of ≥ 12 SNPs showed near-absolute concordance with the clusters derived from the monophyly constrained, tree-based approach (Fig. 2B and C, and Fig. S1). However, for the Thai data set, despite this optimization, a reasonable degree of concordance could only be achieved at a cutoff of ≥22 SNPs, which is exceptionally high, and even then, some discordance still remained (Fig. 3F and G). This, again, suggests that the underlying assumption of pwSNP clustering—i.e., that pwSNP distances can approximate evolutionary relatedness well—breaks down in high-burden, persistently transmitting settings, where the bacterial evolutionary structure lacks clear genetic boundaries, especially at low genetic distances. Consequently, clustering results using low genetic distance thresholds are not robust to the choice of method (Fig. S2). Achieving relatively robust results for the Thai data set would require assuming that cases with meaningful epidemiological links could be separated by at least 22 SNPs, or 26.29 (95% CI: 19.16–41.86) years of evolution, by which point genetic boundaries become clearer. This, however, would severely limit the temporal resolution of the analysis, capturing historical “macro” endemic sub-lineages instead of contemporary transmission clusters. This would, in turn, render the results uninformative for guiding active public health interventions, which are primarily concerned with, and would benefit most from, transmission clusters containing cases that are either linked directly or through a few unsampled intermediate cases.

The limitations of fixed-threshold pwSNP-distance clustering are likely to be further exacerbated by sparse, non-systematic sampling. Sparse, non-systematic sampling could result in many unsampled transmission links, and this likely explains why higher SNP thresholds were required for the Thai data set to yield monophyletic clusters comparable to the Walker et al. (18) data set. Indeed, our analysis revealed that, as the SNP threshold increased, the proportion of samples assigned to multi-sample SNP clusters increased more slowly for the Thai data set than for the UK data set (odds increasing by a factor of 1.538 vs. 1.801 per SNP threshold doubling, respectively; Fig, 2A and 3E), consistent with a lower overall sampling rate. This aligns well with a simulation-based study reporting that lower sampling rates are associated with lower clustering rates and longer terminal branch lengths (47). Nevertheless, our analysis of multi-sample SNP clusters revealed a robust statistical demarcation between monophyletic (optimal and underclustering) and non-monophyletic (paraphyletic, polyphyletic, and para/polyphyletic) clusters. Compared to monophyletic clusters, non-monophyletic clusters were generally associated with larger cluster sizes, greater maximum intra-cluster genetic distances, and broader temporal spans of sample collection (Fig. 4). These metrics may serve as useful empirical indicators for flagging potentially problematic clusters in future pwSNP-distance analyses.

Finally, our results highlight the importance of rigorous sequence quality control, particularly regarding mixed infections and samples with high rates of ambiguous bases. We observed that such samples can form spurious, phylogenetically long-range SNP linkages that artificially bridge multiple clades together (Fig. 1D and 3C). We also noted that mixed samples tended to occupy deep positions within the tree, likely because phylogenetically informative sites in such samples, i.e., sites containing lineage-specific nucleotide variants, will naturally be called polymorphic or ambiguous. In standard phylogenetic reconstruction, these ambiguous bases are typically either treated as missing data or interpreted as having equal likelihood for each possible character, thereby causing mixed samples to be placed deeply within the phylogeny. Conversely, during pwSNP distance calculations, ambiguous bases are usually ignored, leaving only unambiguous bases for comparison. This therefore systematically underestimates the true genetic distance between a mixed sample and its nearest neighbors. Together, these methodological artifacts explain the observed phylogenetically long-range SNP linkages. Indeed, when mixed samples and those with high ambiguous base rates were excluded from analysis, these long-range SNP linkages disappeared and cophenetic correlation improved. These observations strongly underscore the importance of strict filtering for mixed infections prior to conducting clustering analysis to prevent cluster collapse or erroneous cluster assignments.

### Conclusion and future directions

In conclusion, while fixed-threshold pwSNP-distance clustering is computationally efficient and practically useful in low-burden epidemiological contexts, our findings demonstrate that its utility for inferring TB transmission clusters can be limited for high-burden, endemic, sparsely sampled settings. Although our analysis was limited to two distinct data sets, which highlights the need for future evaluations across a wider range of epidemiological settings, our results demonstrate that the interplay of persistent transmission and incomplete sampling can significantly reduce the phylogenetic and epidemiological reliability of the method.

Since the seminal work by Walker et al. (18), the past decade and a half have seen the development of numerous tools for inferring pathogen transmission clusters from WGS data that extend well beyond fixed-threshold pwSNP distance clustering. One such tool is TransCluster (48), which integrates genetic distances with sampling dates under assumed molecular clock and transmission rates to cluster sequences based on the probability that they are separated by a specified number of transmission events directly from a SNP alignment. Although the tool does not explicitly enforce monophyly, compared with pwSNP distance, this provides a more biologically interpretable measure of transmission relatedness and consequently more meaningful transmission clusters. Another tool is TreeCluster (49), which can be used to define clusters directly from a phylogenetic tree under user-defined genetic distance and/or monophyly constraints. The tool offers several clustering algorithms, including the “Max Clade” method, which is conceptually equivalent to the monophyly constrained, tree-based method used in this study. Many reference-free, k-mer-based tools have also been developed for rapid estimation of sequence relatedness, such as MASH (50), PopPUNK (51), and SKA2 (52). A study showed that, when applied to high-quality genome assemblies, these tools can delineate MTBC sublineages accurately and substantially faster than conventional WGS analysis pipelines (53). Although their performance for inferring transmission remains largely unexplored, this study suggests the potential for more computationally efficient genomic epidemiological analyses, which is highly valuable for resource-limited settings.

Beyond cluster delineation, several phylodynamic tools have also been developed to explicitly reconstruct chains of transmission events from molecular data, such as phybreak (54), TransPhylo (55, 56), outbreaker2 (57), and BREATH (58). These tools allow users to integrate genomic data with sampling dates and epidemiological information, such as generation time and sampling proportion distributions, to infer detailed who-infected-whom relationships as well as unsampled cases. This capability is particularly valuable for high-burden settings, where typically only a relatively small proportion of TB cases are sequenced. Consequently, direct transmission links between sampled isolates are less likely to be observed, increasing the risk of underestimating transmission clustering if unsampled cases are not accounted for. Some recently developed tools, such as TransPhylo2 (59), even allow discrete host epidemiological characteristics to be integrated directly into transmission inference, enabling simultaneous estimation of transmission chains and group-specific epidemiological parameters, such as the basic reproduction number and sampling proportion, providing even more useful information for public health planning.

Many of these mentioned downstream transmission inference tools rely, either explicitly or implicitly, on an accurate definition of transmission clusters. Consequently, improvements in cluster delineation will benefit not only cluster-based epidemiological analyses but also the more sophisticated transmission inference methods. Errors introduced during cluster delineation can distort transmission chain reconstruction, bias estimates of transmission between demographic groups, and obscure the identification of transmission risk factors, for example. Our findings reveal that sequence clusters derived using the fixed-threshold pwSNP-distance clustering method can sometimes be phylogenetically inconsistent and therefore provide a less reliable foundation for these downstream analyses than tree-based clustering, particularly in high-burden, endemic settings with sparse, non-systematic sampling, such as Thailand. Consequently, conclusions drawn from such analyses should be interpreted with caution; otherwise, clustering errors may propagate through downstream analyses and ultimately misdirect public health interventions. Wider implementation in routine TB surveillance, however, will require overcoming several practical challenges. Phylogenetic analyses remain more computationally demanding than pwSNP clustering and require greater bioinformatics expertise, particularly for evaluating phylogenetic uncertainty and selecting appropriate clustering criteria. These constraints may be most pronounced in high-burden settings, where computational infrastructure and specialist personnel are often limited. Nonetheless, continued advances in sequencing technologies, computational infrastructure, phylogenetic software, and automated analysis pipelines are expected to continue reducing these barriers. Moreover, many national TB programs are generating increasingly large WGS data sets, and publicly available MTBC genomes continue to accumulate. These expanding genomic resources provide broader evolutionary context, improving phylogenetic inference and further increasing the value of phylogenetically informed analyses, for example, by facilitating the identification of transmission links spanning regions and national borders.

Moving forward, to accurately detect transmission clusters in high-burden settings with persistent transmission chains and incomplete sampling, we therefore recommend transitioning from fixed-threshold pwSNP-distance clustering toward more robust, phylogenetic clustering methods that respect evolutionary descent. Such approaches are likely to produce transmission clusters that are more complete and faithful to the underlying transmission process, thereby providing a stronger foundation for epidemiological investigations and ultimately more informed TB control strategies across diverse epidemiological settings.

## Supplementary Material

Reviewer comments

## Data Availability

All sequence data analyzed in this study were publicly available data. See Tables S1 and S3 for accession numbers of all sequence data analyzed. All code used to generate the figures, tables, and statistical results reported in this study is available at https://github.com/PAiewsakun/Phylogenetic-inconsistency-of-TB-SNP-clusters.

## SUPPLEMENTAL MATERIAL

Fig. S1 msystems.00641-26-s0001.png *t*-SNE visualization of cophenetic distances and clustering concordance in the Walker et al. (2013) data set.

Fig. S2 msystems.00641-26-s0002.png *t*-SNE visualization of cophenetic distances and clustering concordance in the Thai data set.

Fig. S3 msystems.00641-26-s0003.png Phylogenetic visualization of all multi-sample SNP clusters in the Thai data set that were not classified as “optimally monophyletic” using a 5-SNP threshold.

Fig. S4 msystems.00641-26-s0004.png Phylogenetic visualization of all multi-sample SNP clusters in the Thai data set that were not classified as “optimally monophyletic” using a 12-SNP threshold.

Fig. S5 msystems.00641-26-s0005.png Phylogenetic visualization of all multi-sample SNP clusters in the Thai data set that were not classified as “optimally monophyletic” using a 22-SNP threshold.

Supplemental material msystems.00641-26-s0006.pdf Supplemental methods, figure legends, and table legends.

Supplemental tables msystems.00641-26-s0007.xlsx Tables S1 to S6.

OPEN PEER REVIEW reviewer-comments.pdf An accounting of the reviewer comments and feedback.

ASM does not own the copyrights to Supplemental Material that may be linked to, or accessed through, an article. The authors have granted ASM a non-exclusive, world-wide license to publish the Supplemental Material files. Please contact the corresponding author directly for reuse.
