# Supplementary material for: Phylogenetic inconsistency of pairwise SNP clustering for inferring tuberculosis transmission in a high-burden, endemic setting: a case study from Thailand
Source: mSystems. 2026 Sep 14;11(10):e00641-26. doi: 10.1128/msystems.00641-26 (PMC13713008; doi:10.1128/msystems.00641-26)
Supplement: Supplementary file 1 [file msystems.00641-26-s0006.pdf]

Phylogenetic inconsistency of pairwise SNP clustering for inferring tuberculosis transmission in a high-burden, endemic setting: A case study from Thailand

Running title
Phylogenetic inconsistency of TB SNP clusters

Authors
Pakorn Aiewsakun[1,2]*, Walter Samuel Hunter[1,2], Prasit Palittapongarnpim[1,2]

Author affiliations
1. Department of Microbiology, Faculty of Science, Mahidol University, Bangkok, Thailand 10400
2. Pornchai Matangkasombut Center for Microbial Genomics, Department of Microbiology, Faculty of Science, Mahidol University, Bangkok, Thailand 10400

Corresponding author's contact details
Pakorn Aiewsakun: Department of Microbiology, Faculty of Science, Mahidol University, 272, Rama VI Road, Ratchathewi, Bangkok, 10400, Thailand; +66 2 201 5543; pakorn.aie@mahidol.ac.th

Supplementary Methods

Sequence data analysis protocol

Sequence data integrity validation, cleaning, and quality assessment

The integrity of raw FASTQ sequence data was validated using the ‘validate’ function in fqtools v2.0 (1). Paired-end reads were salvaged as single-end reads if only one file passed validation, and if read counts differed between validated forward and reverse files, the forward reads were retained as single-end data for downstream analysis. Trimmomatic v0.40 (2) was used to remove remaining Illumina sequencing adapters (Nextera and TruSeq v2 and v3 paired-end and single-end adapter sequence sets) and low-quality bases, applying a sliding window of 4 bases with a minimum average Phred quality score of 30 (SLIDINGWINDOW:4:30) and discarding reads shorter than 50 bases after trimming (MINLEN:50). Sequence read quality was assessed before and after cleaning using FastQC v0.12.1 (3), and summary reports were generated with MultiQC v1.33 (4).

Read mapping, and calling variant sites with respect to the H37Rv reference genome

Cleaned reads were mapped to the *Mycobacterium tuberculosis* H37Rv reference genome assembly (NC_000962.3) using BWA-MEM v0.7.19-r1273 (5), limiting the occurrence of highly repetitive seeds during alignment to ≤100 (-c 100), marking shorter split alignments as secondary (-M), and requiring a minimum alignment score of 50 for reported mappings (-T 50). Duplicate reads were marked using Picard v3.4.0 (6) via the ‘MarkDuplicates’ function in the Genome Analysis Toolkit (GATK) v4.6.2.0 (7–9), retaining flagged duplicate reads in the output (--REMOVE_DUPLICATES false) and allowing lenient validation of read records (--VALIDATION_STRINGENCY LENIENT). Read mapping statistics were computed using the ‘coverage’ function in SAMtools v1.23 (10, 11) with default settings. Sample-wise variant calling was performed using the ‘HaplotypeCaller’ function in GATK v4.6.2.0 (7–9), generating sample-wise gVCF files (-ERC GVCF), requiring a minimum base quality score of 20 (--min-base-quality-score 20) to be reported, and setting ploidy to 2 (-ploidy 2) to enable detection of within-sample polymorphic sites. To compute the number of polymorphic sites for each sample, detected heterozygous sites were extracted from the single-sample gVCF file using the ‘query’ function in BCFtools v1.23 (10, 11). Minor allele frequencies (MAFs) were calculated from allele depth values reported in the gVCF files. Sites were considered as polymorphic if at least two alleles were supported by sequence reads, and had a minor allele frequency ≥0.05. The generated gVCF files were also analysed to determine samples’ lineage, and mixed infection status (see below).

Sample and variant site quality control, and joint genotyping

Sample-level quality control excluded samples meeting any of the following criteria: average mapping depth <10×, genome coverage <90%, >10,000 polymorphic sites, or an average MAF >0.4. Remaining sample-wise gVCF files were hard-filtered using the ‘filter’ function from BCFtools v1.23 (10, 11) to exclude sites with depth <10 (-g 10) and to mask single nucleotide polymorphism (SNP) sites within 10 base pairs of an indel (-e 'INFO/DP<10'). Joint genotyping was then performed using the remaining, clean gVCF files as input with ‘GenomicsDBImport’ and ‘GenotypeGVCFs’ functions from GATK v4.6.2.0 (7–9) with ploidy set to 2 (-ploidy 2), generating a multi-sample VCF file.

To clean the data further, we filtered the resulting multi-sample VCF file using the ‘SelectVariants’ function from GATK v4.6.2.0 (7–9) in order to retain only SNP variants (-select-type SNP) and to restrict calls to biallelic sites only (--restrict-alleles-to BIALLELIC). Low-confidence SNP variants were further excluded using the ‘VariantFiltration’ function from GATK v4.6.2.0 (7–9), excluding sites with variant confidence normalised by depth <2.0 (QD < 2.0) or a root mean square mapping quality <40 (MQ < 40.0). Sites located within refined low-confidence or low pileup mappability regions (12), and genes known to be associated with drug resistance (310,371 unique positions in total) were masked using the ‘view’ function from BCFtools v1.23 (10, 11) to minimise potential mapping artefacts and false clustering signals arising from convergent evolution.

Multiple sequence alignment construction, and further variant site filtering

The clean multi-sample VCF file was converted into a raw multi-sequence alignment (MSA) ignoring marked / masked sites. Bases at polymorphic sites were genotyped as ambiguous bases using IUPAC nucleotide codes, while uncalled or missing positions were genotyped as 'N'. A custom three-pass script was used to further filter the MSA. In the first pass, columns containing >80% missing or ambiguous bases (i.e., non-standard bases) were removed. In the second pass, samples containing >20% remaining non-standard bases were excluded from the alignment. In the third pass, fully conserved invariant sites (ignoring remaining missing or ambiguous bases) were removed from the MSA to generate a SNP-only alignment.

Phylogenetics reconstruction

A maximum likelihood phylogeny was constructed using IQ-TREE v3.0.1 (13) with the best-fit nucleotide substitution model automatically selected by ModelFinder (14) under the default Bayesian information criterion. Invariant A/C/G/T base counts were provided to the program directly via the -fconst argument to correct base ascertainment bias. These counts were computed by counting the number of native A/C/G/T bases in the reference genome at positions not present in the filtered MSA, and adding these counts to the number of A/C/G/T invariant sites removed during the third pass of MSA filtering. The calculation excluded reference sites containing non-standard bases, sites located within masked genomic regions, and positions excluded from the MSA due to excessive non-standard bases.

Pairwise SNP distance calculation and clustering analysis

Pairwise SNP distances were computed from the filtered MSA using snp-dists v1.2.0 (15) with default settings, considering nucleotide pairs containing standard A/C/G/T bases only. An undirected network graph was constructed from the obtained distances using the igraph v2.1.4 package in R v4.4.2 (16). Samples were considered 'SNP linked' if their pairwise SNP distance was less than or equal to predefined SNP distance thresholds, and 'SNP clusters' were defined as connected components within the resulting graph.

Lineage assignment, and mixed infection detection

Lineages were assigned with mtbtyper v1.0.0 (17) using all available SNP schemes (--all_schemes) and the GATK-generated single-sample gVCF files as input. For samples with a median read mapping depth ≥5×, 'mixed samples', defined as containing multiple lineages where the frequency of the minor lineage was ≥ 0.05, were identified using TBtypeR v25.1.0 (18) running in R v4.4.2 (16). As recommended by the developers, VCF files generated by BCFtools v1.23 (10, 11) were used as input for this analysis instead of the GATK-generated gVCF files. Specifically, the 'mpileup' function was first run on the samples' read-mapping BAM alignment files with duplicate reads masked to generate genotype likelihoods at each genomic position, requiring a minimum read mapping quality of 20 (-q 20), a minimum base quality score of 20 (-Q 20), and a maximum read depth of 200 per position (-d 200). Variant sites were then called using the 'call' function, retaining all alleles (-A), applying the multiallelic calling model (-m), and specifying a prior mutation rate of 10^{-2} (--prior 1e-2).

Supplementary figures

Figure S1 t-SNE visualisation of cophenetic distances and clustering concordance in the Walker et al. (2013) dataset. The estimated cophenetic distance matrix is projected into a two-dimensional space using t-distributed Stochastic Neighbor Embedding (t-SNE). Each grey point represents an individual sample. The t-SNE analysis was parameterised with a perplexity of 15 and a maximum of 1,000 iterations. To prevent coordinate calculation errors caused by identical samples (i.e., zero cophenetic distance), a minor spatial jitter was applied to the resulting coordinates. Concave hulls denote the boundaries of clusters containing two or more samples: red hulls, pwSNP clusters; blue hulls, tree-based clusters. The two panels display clustering assignments evaluated at two different SNP thresholds: 5-SNP (top) and 12-SNP (bottom). Only multi-sample clusters are shown. The underlying t-SNE coordinates are fixed across both panels, allowing for direct comparison of how incrementally relaxing the SNP threshold alters clustering results.

Figure S2 t-SNE visualisation of cophenetic distances and clustering concordance in the Thai dataset. t-SNE analysis and plotting conventions are identical to those described in **Figure S1**. The three panels display clustering assignments evaluated at three different SNP thresholds: 5-SNP (top), 12-SNP (middle), and 22-SNP (bottom).

Figure S3 Phylogenetic visualisation of all multi-sample SNP clusters in the Thai dataset that were not classified as "optimally monophyletic" using a 5-SNP threshold. All multi-sample SNP clusters that were not classified as "optimally monophyletic" (i.e., under-clustered monophyletic, paraphyletic, para/polyphyletic, or polyphyletic) are shown. For each panel, the depicted tree shows the 'extended clade' (defined by the most recent common ancestor of all SNP cluster members together with members of any overlapping tree-based clusters). Tip colours denote sample status: SNP cluster members (green), intruders (samples within the base clade but outside the SNP cluster; red), and outruders (samples outside the base clade but within the extended clade; blue). Arcing lines connect sample pairs exhibiting a pwSNP distance of 5 or fewer SNPs. Shaded clades highlight the tree-based clusters, colour-coded by their association with the target SNP cluster (green: sharing at least one member with the SNP cluster; red: sharing no members with the SNP cluster, but belonging to the same extended clade). SNP cluster properties are summarised in the subtitle of each panel.

Figure S4 Phylogenetic visualisation of all multi-sample SNP clusters in the Thai dataset that were not classified as "optimally monophyletic" using a 12-SNP threshold. Plotting conventions, including tip statuses, shading, and arcing pwSNP linkages (representing distances of 12 or fewer SNPs), are identical to those described in **Figure S3**.

Figure S5 Phylogenetic visualisation of all multi-sample SNP clusters in the Thai dataset that were not classified as "optimally monophyletic" using a 22-SNP threshold. Plotting conventions, including tip statuses, shading, and arcing pwSNP linkages (representing distances of 22 or fewer SNPs), are identical to those described in **Figure S3**.

Supplementary tables

Table S1 Outputs from the sequence data analysis pipeline for the Walker et al. (2013) dataset. Collection years are provided where available.

Table S2 pwSNP-based clustering results using thresholds ranging between 1 to 25 SNPs with the corresponding tree-based clustering assignments evaluated using optimal cophenetic distances for the Walker et al. (2013) dataset.

Table S3 Outputs from the sequence data analysis pipeline for the Thai dataset. Collection years are provided where available.

Table S4 pwSNP-based clustering results using thresholds ranging between 1 to 25 SNPs with the corresponding tree-based clustering assignments evaluated using optimal cophenetic distances for the Thai dataset.

Table S5 Comparison of multi-sample SNP cluster properties across phylogenetic classes. For each evaluated property and SNP threshold (5, 12, and 22 SNPs), the table presents the number of clusters (n), median, interquartile range (q1-q3), and mean rank for each phylogenetic class (visualised in **Figure 4**). Global variation across classes was assessed using the Kruskal-Wallis test. All properties showed significant global variation (KW p-value < 0.05). Post-hoc pairwise comparisons were subsequently conducted using Dunn's test with the Benjamini-Hochberg multiple-testing correction procedure (pairwise p values are reported in **Table S6**). The resulting statistical groupings are denoted by a compact letter display in the 'stat_group' column, wherein classes sharing at least one common letter do not differ significantly at an alpha level of 0.05.

Table S6 Pairwise p-values from post-hoc Dunn's tests conducted following the global Kruskal-Wallis significance tests reported in Table S5. Adjusted p-values were calculated using the Benjamini-Hochberg procedure to control the false discovery rate.

References

1. Droop AP. 2016. fqtools: An efficient software suite for modern FASTQ file manipulation. Bioinformatics 32:1883–1884.
2. Bolger AM, Lohse M, Usadel B. 2014. Trimmomatic: A flexible trimmer for Illumina sequence data. Bioinformatics 30:2114–2120.
3. Andrews S. 2018. FastQC A quality control tool for high throughput sequence data. Babraham Bioinformatics. https://www.bioinformatics.babraham.ac.uk/projects/fastqc/.
4. Ewels P, Magnusson M, Lundin S, Käller M. 2016. MultiQC: Summarize analysis results for multiple tools and samples in a single report. Bioinformatics 32:3047–3048.
5. Vasimuddin M, Misra S, Li H, Aluru S. 2019. Efficient architecture-aware acceleration of BWA-MEM for multicore systems, p. 314–324. *In* IEEE International Parallel and Distributed Processing Symposium (IPDPS).
6. Broad Institute. 2019. Picard tools (3.4.0).
7. McKenna A, Hanna M, Banks E, Sivachenko A, Cibulskis K, Kernytsky A, Garimella K, Altshuler D, Gabriel S, Daly M, DePristo MA. 2010. The Genome Analysis Toolkit: a MapReduce framework for analyzing next-generation DNA sequencing data. Genome Res 20:1297–1303.
8. DePristo MA, Banks E, Poplin R, Garimella KV, Maguire JR, Hartl C, Philippakis AA, del Angel G, Rivas MA, Hanna M, McKenna A, Fennell TJ, Kernytsky AM, Sivachenko AY, Cibulskis K, Gabriel SB, Altshuler D, Daly MJ. 2011. A framework for variation discovery and genotyping using next-generation DNA sequencing data. Nat Genet 43:491–498.
9. Van der Auwera GA, Carneiro MO, Hartl C, Poplin R, del Angel G, Levy-Moonshine A, Jordan T, Shakir K, Roazen D, Thibault J, Banks E, Garimella KV, Altshuler D, Gabriel S, DePristo MA. 2013. From fastQ data to high-confidence variant calls: The genome analysis toolkit best practices pipeline. Current Protocols in Bioinformatics 43:11.10.1-11.10.33.
10. Danecek P, McCarthy SA. 2017. BCFtools/csq: haplotype-aware variant consequences. Bioinformatics 33:2037–2039.
11. Danecek P, Bonfield JK, Liddle J, Marshall J, Ohan V, Pollard MO, Whitwham A, Keane T, McCarthy SA, Davies RM, Li H. 2021. Twelve years of SAMtools and BCFtools. GigaScience 10:giab008.
12. Marin M, Vargas R, Harris M, Jeffrey B, Epperson LE, Durbin D, Strong M, Salfinger M, Iqbal Z, Akhundova I, Vashakidze S, Crudu V, Rosenthal A, Farhat MR. 2022. Benchmarking the empirical accuracy of short-read sequencing across the *M. tuberculosis* genome. Bioinformatics 38:1781–1787.
13. Wong T, Ly-Trong N, Ren H, Baños H, Roger A, Susko E, Bielow C, De Maio N, Goldman N, Hahn M, Huttley G, Lanfear R, Minh BQ. 2025. IQ-TREE 3: Phylogenomic inference software using complex evolutionary models https://doi.org/10.32942/X2P62N.
14. Kalyaanamoorthy S, Minh BQ, Wong TKF, Von Haeseler A, Jermiin LS. 2017. ModelFinder: Fast model selection for accurate phylogenetic estimates. Nature Methods 14:587–589.
15. Torsten Seemann, Fabian Klötzl, Andrew J Page. 2025. snp-dists (1.2.0).
16. R Core Team. 2014. R: A Language and environment for statistical computing. R Foundation for Statistical Computing 1:409.
17. Thawornwattana Y, Jaemsai B. 2022. mtbtyper (1.0.0).
18. Munro JE, Coussens AK, Bahlo M. 2025. TBtypeR: Sensitive detection and sublineage classification of *Mycobacterium tuberculosis* complex mixed-strain infections. Commun Biol 8:260.
