# Supplementary material for: Phylogenetic inconsistency of pairwise SNP clustering for inferring tuberculosis transmission in a high-burden, endemic setting: a case study from Thailand
Source: mSystems. 2026 Sep 14;11(10):e00641-26. doi: 10.1128/msystems.00641-26 (PMC13713008; doi:10.1128/msystems.00641-26)
Supplement: Supplementary file 2 [file reviewer-comments.pdf]

ASM | mSystems

Phylogenetic inconsistency of pairwise SNP clustering for inferring tuberculosis transmission in a high-burden, endemic setting: A case study from Thailand

Pakorn Aiewsakun, Walter Hunter, and Prasit Palittapongarnpim

Corresponding Author(s): Pakorn Aiewsakun, Mahidol University Faculty of Science

**Review Timeline:**		
	Submission Date:	May 16, 2026
	Editorial Decision:	July 18, 2026
	Revision Received:	August 4, 2026
	Accepted:	August 19, 2026

Editor: Sophie Darch

Reviewer(s): Disclosure of reviewer identity is with reference to reviewer comments included in decision letter(s). The following individuals involved in review of your submission have agreed to reveal their identity: Paul Keim (Reviewer #2)

Transaction Report:

(Note: With the exception of the correction of typographical or spelling errors that could be a source of ambiguity, letters and reports are not edited. The original formatting of letters and referee reports may not be reflected in this compilation.)

DOI: https://doi.org/10.1128/msystems.00641-26

Re: mSystems00641-26 (Phylogenetic inconsistency of pairwise SNP clustering for inferring tuberculosis transmission in a high-burden, endemic setting: A case study from Thailand)

Dear Dr. Pakorn Aiewsakun:

Thank you for the privilege of reviewing your work. Below you will find my comments, instructions from the mSystems editorial office, and the reviewer comments.

Please return the manuscript within 60 days; if you cannot complete the modification within this time period, please contact me. If you do not wish to modify the manuscript and prefer to submit it to another journal, notify me immediately so that the manuscript may be formally withdrawn from consideration by mSystems.

Revision Guidelines
To submit your modified manuscript, log into the submission site at https://msystems.msubmit.net/cgi-bin/main.plex. Go to Author Tasks and click the appropriate manuscript title to begin. The information you entered when you first submitted the paper will be displayed; update this as necessary. Note the following requirements:

• Upload point-by-point responses to the issues raised by the reviewers in a file named "Response to Reviewers," NOT in your cover letter.
• Upload a compare copy of the manuscript (without figures) as a "Marked-Up Manuscript" file.
• Upload a clean .DOC/.DOCX version of the revised manuscript and remove the previous version.
• Each figure must be uploaded as a separate, editable, high-resolution file (TIFF or EPS preferred), and any multipanel figures must be assembled into one file.
• Any supplemental material intended for posting by ASM should be uploaded with their legends separate from the main manuscript. You can combine all supplemental material into one file (preferred) or split it into a maximum of 10 files with all associated legends included.

For complete guidelines on revision requirements, see our Submission and Review Process webpage. Submission of a paper that does not conform to guidelines may delay acceptance of your manuscript.

Data availability: ASM policy requires that data be available to the public upon online posting of the article, so please verify all links to sequence records, if present, and make sure that each number retrieves the full record of the data. If a new accession number is not linked or a link is broken, provide mSystems production staff with the correct URL for the record. If the accession numbers for new data are not publicly accessible before the expected online posting of the article, publication may be delayed; please contact production staff (mSystems@asmusa.org) immediately with the expected release date.

Publication Fees: For information on publication fees and which article types are subject to charges, visit our website. If your manuscript is accepted for publication and any fees apply, you will be contacted separately about payment during the production process; please follow the instructions in that e-mail. Arrangements for payment must be made before your article is published.

ASM Membership: Corresponding authors may join or renew ASM membership to obtain discounts on publication fees. Need to upgrade your membership level? Please contact Customer Service at Service@asmusa.org.

The ASM Journals program strives for constant improvement in our submission and publication process. Please tell us how we can improve your experience by taking this quick Author Survey.

Thank you for submitting your paper to mSystems.

Sincerely,
Sophie Darch
Editor
mSystems

Reviewer #2 (Comments for the Author):

This is an important paper that I hope has an impact on the tuberculosis community. The work is rigorous and the paper well written. I started reading it with multiple pitfalls in mind and the authors addressed them all: sequence quality, mixed genotypes in a single person, endemic vs. non endemic zone sampling, and the variability in transmission dynamics. Not to belittle this paper, but the conclusions will be very obvious to the phylogenomics community. The fact that these are not obvious to the

epidemiologists who would use a simple SNP threshold, makes this paper even more important. The mostly non existent molecular clock is a great illustration of why "one shoe does not fit all" in molecular epidemiology. Good job. The epidemiological consequences of using only a 12 or 25 SNP threshold to identify transmission clusters are bad. This clarity and rigor of this paper will help guide the application of WGS.

Reviewer #3 (Comments for the Author):

This manuscript presents a careful and timely comparison of pairwise SNP (pwSNP)-distance clustering and phylogeny-based clustering methods for delineating Mycobacterium tuberculosis transmission clusters using two contrasting epidemiologic settings: a low-burden setting with limited ongoing transmission and a high-burden endemic setting. The analyses are thorough, and the inclusion of two distinct datasets provides a valuable empirical assessment of how clustering approaches perform under different transmission scenarios. Overall, the study contributes useful evidence supporting the transition from fixed SNP-threshold clustering toward phylogeny-informed approaches that better reflect evolutionary relationships.

That said, I found that the central conclusion-that phylogenetic clustering is generally more robust than fixed SNP-threshold clustering-is largely consistent with current understanding in the field. The primary value of the manuscript lies in its comprehensive case studies rather than in introducing a fundamentally new methodological insight. I therefore believe the manuscript would have greater impact if the authors more clearly positioned their findings within the rapidly evolving landscape of WGS-based transmission inference.

In particular, I encourage the authors to expand the Discussion in the following ways:

1. Provide a broader overview of contemporary clustering and transmission inference methods. The discussion focuses primarily on pairwise SNP-distance and tree-based clustering, but several complementary approaches have emerged in recent years. A concise discussion of these approaches would help readers better understand where the proposed recommendations fit within the current methodological landscape.
2. Expand the public health implications. The manuscript would benefit from a more detailed discussion of how adopting phylogenetic clustering could influence tuberculosis surveillance and control programs. Are there computational or personnel barriers that may limit adoption in high-burden settings? Discussing these issues would substantially increase the translational relevance of the work.

<u>Point-by-point response to reviewer's comments</u>

Reviewer #2's comments:
This is an important paper that I hope has an impact on the tuberculosis community. The work is rigorous and the paper well written. I started reading it with multiple pitfalls in mind and the authors addressed them all: sequence quality, mixed genotypes in a single person, endemic vs. non endemic zone sampling, and the variability in transmission dynamics. Not to belittle this paper, but the conclusions will be very obvious to the phylogenomics community. The fact that these are not obvious to the epidemiologists who would use a simple SNP threshold, makes this paper even more important. The mostly non existent molecular clock is a great illustration of why "one shoe does not fit all" in molecular epidemiology. Good job. The epidemiological consequences of using only a 12 or 25 SNP threshold to identify transmission clusters are bad. This clarity and rigor of this paper will help guide the application of WGS.
Reply: We thank the reviewer for their very positive comments.

Reviewer #3's comments:

This manuscript presents a careful and timely comparison of pairwise SNP (pwSNP)-distance clustering and phylogeny-based clustering methods for delineating Mycobacterium tuberculosis transmission clusters using two contrasting epidemiologic settings: a low-burden setting with limited ongoing transmission and a high-burden endemic setting. The analyses are thorough, and the inclusion of two distinct datasets provides a valuable empirical assessment of how clustering approaches perform under different transmission scenarios. Overall, the study contributes useful evidence supporting the transition from fixed SNP-threshold clustering toward phylogeny-informed approaches that better reflect evolutionary relationships.

That said, I found that the central conclusion-that phylogenetic clustering is generally more robust than fixed SNP-threshold clustering-is largely consistent with current understanding in the field. The primary value of the manuscript lies in its comprehensive case studies rather than in introducing a fundamentally new methodological insight. I therefore believe the manuscript would have greater impact if the authors more clearly positioned their findings within the rapidly evolving landscape of WGS-based transmission inference.

Reply: We thank the reviewer for their very positive and constructive comments. The specific points raised are addressed below. Line numbers refer to the tracked-change version of the revised manuscript.

In particular, I encourage the authors to expand the Discussion in the following ways:

1. Provide a broader overview of contemporary clustering and transmission inference methods. The discussion focuses primarily on pairwise SNP-distance and tree-based clustering, but several complementary approaches have emerged in recent years. A concise discussion of these approaches would help readers better understand where the proposed recommendations fit within the current methodological landscape.

Reply: We thank the reviewer for this suggestion. We have now substantially expanded the final paragraph of the Discussion into a new ***Conclusion and future direction*** section to provide a broader overview of contemporary clustering and transmission inference methods as suggested by the reviewer (lines 586-640). As this discussion naturally overlaps with the public health implications of our findings, the text also partially addresses the reviewer's second point below.

> ## Conclusion and future direction
>
> *In conclusion, while fixed-threshold pwSNP-distance clustering ~~with a fixed threshold~~ is computationally efficient and practically useful in low-burden epidemiological contexts, our findings demonstrate that its utility for inferring TB transmission clusters can be limited for high-burden, endemic, sparsely sampled settings. Although our analysis was limited to ~~focused only on~~ two distinct datasets, which highlights the need for future evaluations across a wider range of epidemiological settings, ~~other diverse regions,~~ our results ~~clearly~~ demonstrate that the interplay of persistent transmission and incomplete sampling can significantly reduce the phylogenetic and epidemiological reliability of the method.*
>
> *Since the seminal work by Walker et al. (2013) (18), the past decade and a half have seen the development of numerous tools for inferring pathogen transmission clusters from WGS data that extend well beyond fixed-threshold pwSNP distance clustering. One such tool is TransCluster (48), which integrates genetic distances with sampling dates under assumed molecular clock and transmission rates to cluster sequences based on the probability that they are separated by a specified number of transmission events directly from a SNP alignment. Although the tool does not explicitly enforce monophyly, compared with pwSNP distance, this provides a more biologically interpretable measure of transmission relatedness, and consequently more meaningful transmission clusters. Another tool is TreeCluster (49),*

which can be used to define clusters directly from a phylogenetic tree under user-defined genetic distance and/or monophyly constraints. The tool offers several clustering algorithms, including the "Max Clade" method, which is conceptually equivalent to the monophyly-constrained, tree-based method used in this study. Many reference-free, k-mer-based tools have also been developed for rapid estimation of sequence relatedness, such as MASH (50), PopPUNK (51), and SKA2 (52). A study showed that, when applied to high-quality genome assemblies, these tools can delineate MTBC sublineages accurately and substantially faster than conventional WGS analysis pipelines (53). Although their performance for inferring transmission remains largely unexplored, this study suggests the potential for more computationally efficient genomic epidemiological analyses, which is highly valuable for resource-limited settings.

Beyond cluster delineation, several phylodynamic tools have also been developed to explicitly reconstruct chains of transmission events from molecular data, such as phybreak (54), TransPhylo (55, 56), outbreaker2 (57), and BREATH (58). These tools allow users to integrate genomic data with sampling dates, and epidemiological information, such as generation time and sampling proportion distributions, to infer detailed who-infected-whom relationships as well as unsampled cases. This capability is particularly valuable for high-burden settings, where typically only a relatively small proportion of TB cases are sequenced. Consequently, direct transmission links between sampled isolates are less likely to be observed, increasing the risk of underestimating transmission clustering if unsampled cases are not accounted for. Some recently developed tools, such as TransPhylo2 (59), even allow discrete host epidemiological characteristics to be integrated directly into transmission inference, enabling simultaneous estimation of transmission chains and group-specific epidemiological parameters, such as the basic reproduction number and sampling proportion, providing even more useful information for public health planning.

Many of these mentioned downstream transmission inference tools rely, either explicitly or implicitly, on an accurate definition of transmission clusters. Consequently, improvements in cluster delineation will benefit not only cluster-based epidemiological analyses but also the more sophisticated transmission inference methods. Errors introduced during cluster delineation can distort transmission chain reconstruction, bias estimates of transmission between demographic groups, and obscure the identification of transmission risk factors, for example. Our findings reveal that sequence clusters derived using the fixed-threshold pwSNP-distance clustering method can sometimes be phylogenetically inconsistent, and therefore provide a less reliable foundation for these downstream analyses than tree-based clustering, particularly in high-burden, endemic settings with sparse, non-systematic sampling, such as Thailand. Consequently, conclusions drawn from such analyses should be interpreted with caution, otherwise clustering errors may propagate through downstream analyses, and ultimately misdirect public health interventions…

2. Expand the public health implications. The manuscript would benefit from a more detailed discussion of how adopting phylogenetic clustering could influence tuberculosis surveillance and control programs. Are there computational or personnel barriers that may limit adoption in high-burden settings? Discussing these issues would substantially increase the translational relevance of the work.
Reply: We thank the reviewer for this suggestion. As noted above, the translational public health implications of our findings are now discussed in the new ***Conclusion and future direction*** section (see above). In addition, as specifically suggested by the reviewer in this comment, we have now also discussed the practical barriers to implementing phylogenetically informed approaches in routine TB surveillance in this section, including computational requirements and bioinformatics expertise. We

also discuss how the growing availability of national and public WGS datasets further increases the value of phylogenetically informed analyses for routine surveillance and cross-border transmission investigations (lines 640-660):

> *…Wider implementation in routine TB surveillance, however, will require overcoming several practical challenges. Phylogenetic analyses remain more computationally demanding than pwSNP clustering, and require greater bioinformatics expertise, particularly for evaluating phylogenetic uncertainty and selecting appropriate clustering criteria. These constraints may be most pronounced in high-burden settings, where computational infrastructure and specialist personnel are often limited. Nonetheless, continued advances in sequencing technologies, computational infrastructure, phylogenetic software, and automated analysis pipelines are expected to continue reducing these barriers. Moreover, many national TB programmes are generating increasingly large WGS datasets, and publicly available MTBC genomes continue to accumulate. These expanding genomic resources provide broader evolutionary context, improving phylogenetic inference and further increasing the value of phylogenetically informed analyses, for example, by facilitating the identification of transmission links spanning regions and national borders.*
>
> *Moving forward, to accurately detect transmission clusters in ~~these complex~~ high-burden settings with persistent transmission chains and incomplete sampling, we therefore recommend transitioning from fixed-threshold pwSNP-distance clustering toward more robust, phylogenetic clustering methods that respect evolutionary descent. Such approaches are likely to produce transmission clusters that are more complete and faithful to the underlying transmission process, thereby providing a stronger foundation for epidemiological investigations, and ultimately more informed TB control strategies across diverse epidemiological settings.*

Re: mSystems00641-26R1 (Phylogenetic inconsistency of pairwise SNP clustering for inferring tuberculosis transmission in a high-burden, endemic setting: A case study from Thailand)

Dear Dr. Pakorn Aiewsakun:

Your manuscript has been accepted, and I am forwarding it to the ASM production staff for publication. Your paper will first be checked to make sure all elements meet the technical requirements. ASM staff will contact you if anything needs to be revised before copyediting and production can begin. Otherwise, you will be notified when your proofs are ready to be viewed.

Data Availability: ASM policy requires that data be available to the public upon online posting of the article, so please verify all links to sequence records, if present, and make sure that each number retrieves the full record of the data. If a new accession number is not linked or a link is broken, provide production staff with the correct URL for the record. If the accession numbers for new data are not publicly accessible before the expected online posting of the article, publication may be delayed; please contact ASM production staff immediately with the expected release date.

Publication Fees: For information on publication fees and which article types have charges, please visit our website. We have partnered with Copyright Clearance Center (CCC) to collect author charges. If fees apply to your paper, you will receive a message from no-reply@copyright.com with further instructions. For questions related to paying charges through RightsLink, please contact CCC at ASM_Support@copyright.com or toll free at +1-877-622-5543. CCC makes every attempt to respond to all emails within 24 hours.

ASM Membership: Corresponding authors may join or renew ASM membership to obtain discounts on publication fees. Need to upgrade your membership level? Please contact Customer Service at Service@asmusa.org.

Embargo Policy: A press release may be issued as soon as the manuscript is posted on the mSystems Latest Articles webpage. The corresponding author will receive an email with the subject line "ASM Journals Author Services Notification" when the article is available online.

Cover Image Submissions: If you would like to submit a potential Cover Image, please email a file and a short legend to msystems@asmusa.org. Please note that we can only consider images that (i) the authors created or own and (ii) have not been previously published. By submitting, you agree that the image can be used under the same terms as the published article. Image File requirements: TIF/EPS, 7.5 inches wide by 8.25 inches tall (at least 2,250 pixels wide by 2,475 pixels tall), minimum 300 dpi resolution (600 dpi preferred), RGB, and no figure elements, e.g., arrows or panel labels. The legend should be a short description of the image, 1-2 sentences recommended. Please use the ASM Journals Cover Image Sizing Tool (https://journals.asm.org/pb-assets/html/asm-cover-sizing-tool.html) to ensure that your proposed cover image meets our size requirements.

Thank you for submitting your paper to mSystems.

Sincerely,
Sophie Darch
Editor
mSystems
